# Improved draft genome of *Ignatzschineria larvae* DSM 13226 via hybrid assembly

**DOI:** 10.1128/mra.00428-24

**Published:** 2024-09-30

**Authors:** Emily G. Cantrell, Zachary M. Burcham

**Affiliations:** 1 Department of Microbiology, University of Tennessee, Knoxville, Tennessee, USA; University of Maryland School of Medicine, Baltimore, Maryland, USA

**Keywords:** genomes, blow fly, decomposition, wound, maggot, *Wohlfahrtia*, hybrid assembly, Illumina, Nanopore

## Abstract

*Ignatzschineria larvae* is studied for its role in decomposition and disease ecology; however, the type strain reference genome remains fragmented. The current reference genome consists of 61 contigs calculated at 82.18% complete with 10.98% contamination. Here, we announce the hybrid genome assembly as an improved single contig.

## ANNOUNCEMENT

*Ignatzschineria larvae* is a Gram-negative bacterium in the *Gammaproteobacteria* class. *I. larvae* cells are rod-shaped, non-motile, and non-spore-forming. *I. larvae* was first isolated from the larvae of the parasitic fly *Wohlfahrtia magnifica* (1). *Ignatzschineria* species are detected in blow flies (2)*,* which are found globally (3), and associated with decomposing organic matter (4, 5). *I. larvae* has also been studied for its role in open-wound disease ecology (6). *Ignatzschineria larvae* strain DSM 13226 is the type strain and NCBI reference genome (NCBI RefSeq: GCF_000510805.1). However, the genome assembly of *I. larvae* DSM 13226 was created using only short-read sequencing technology and remains fragmented. The NCBI RefSeq reference genome size is 2.5 Mb in 61 contigs within 52 scaffolds and calculated at 82.18% complete with 10.98% contamination.

To improve this genome assembly, we obtained the same *I. larvae* DSM 13226 strain from the Leibniz Institute DSMZ-German Collection of Microorganisms in February of 2023 used to assemble the original NCBI reference genome. Twenty percent glycerol with brain heart infusion (BHI) broth freezer stock was stored at −70°C, used to streak for isolation onto a BHI agar plate, and grown at 30°C overnight. An individual colony was picked and added to 5 mL of BHI broth and incubated aerobically overnight at 30°C shaking at 200 RPM. The overnight culture was centrifuged at 6,000 × *g* relative centrifugal force (RCF) to pellet the cells. The supernatant was removed, and the cell pellet was resuspended in 500 µL of DNA/RNA Shield (Zymo Research). The preserved cells were shipped to Plasmidsaurus for gDNA extraction using the Quick-DNA Fungal/Bacteria Miniprep Kit (Zymo Research) then subsequent hybrid Oxford Nanopore Technology (ONT) long-read + Illumina short-read bacterial whole genome sequencing. The manufacturer’s recommended protocols were followed unless otherwise stated.

An amplification-free long-read sequencing library was prepared with the ONT Rapid Barcoding Kit 96 V14 without DNA ligation, shearing, or size selection. ONT long-read sequencing was performed on the PromethION P24 using the R10.4.1 flow cell and ont-doradod-for-promethion v7.1.4 base calling software on the “super accurate” mode. ONT reads below a *Q*-score of 10 were discarded, and adapters were trimmed using MinKnow. ONT reads were further filtered by the removal of the bottom 5% quality reads using Filtlong v0.2.1 and downsampled to 100× coverage. A draft consensus ONT assembly was generated using Flye v2.9.1 (7) and polished with the downsampled ONT reads using Medaka v1.8.0. An Illumina short-read sequencing library was prepared with the ExpressPlex 96 Library Prep Kit (SeqWell) and sequenced on the NextSeq2000 using paired-end 2 × 150 bp chemistry. Illumina forward and reverse short reads were aligned to the consensus ONT assembly using BWA-MEM, which clips low-quality sequences from the ends of the reads. The BWA-MEM alignment and consensus ONT assembly were used to generate the final, polished hybrid assembly with Polypolish v0.5.0 (8). The hybrid assembly was annotated with NCBI Prokaryotic Genome Annotation Pipeline v6.7 via the best-placed reference protein set (GeneMarkS-2+) annotation method (9), quality checked with CheckM v1.2.2 (10), and the species verified as *I. larvae* DSM 13226 with Mash v2.3 (11) against NCBI RefSeq genomes. Circularization was checked by prepending the last 500 bp of the contig to the start to create a stitched contig. Both sequencing data sets were aligned to the stitched contig using minimap2. The alignments were merged, and coverage was visualized at the stitched site in the Integrative Genomics Viewer.

Here, we provide the improved, near-complete genome sequence of *Ignatzschineria larvae* DSM 13226 as a single contig (Table 1). The total base pair from long-read sequencing was 558,530,493 bp from 114,405 total reads (max length: 92,756 bp, rN50: 8,831 bp) at 217× raw coverage. The total base pair from short-read sequencing was 414,631,932 bp from 2,607,748 total reads. The hybrid assembly is one contig with a size of 2,563,334 bp, 40.5% GC content, and 84.1% coding density at 99.3× coverage. Annotation predicted 56 tRNAs, 6 complete rRNA sets, 4 ncRNAs, 2 CRISPR arrays, 2,127 total genes, and 2,049 total coding sequences (CDSs). CheckM genome quality calculated 100% completeness and 0% contamination, but circularization could not be verified due to a single coverage gap at the contig stich site. However, the hybrid assembly presented here still represents a significant improvement of the *I. larvae* DSM 13226 reference genome.

**TABLE 1:** Summary of assembly statistics between the *I. larvae* DSM 13226 NCBI reference genome GCF_000510805.1 and improved hybrid assembly CP150637.1

Assembly	Genome size	No. contigs	Contig N50	Total genes	Total CDSs	Completeness/contamination
GCF_000510805.1	2.5 Mb	61	0.0895 Mb	2,103	2,033	82.18/10.98%
CP150637.1	2.56 Mb	1	2.56 Mb	2,127	2,049	100/0%

## Data Availability

The Ignatzschineria larvae DSM 13226 hybrid assembly genome sequence has been deposited in NCBI GenBank under accession no. CP150637. The version described in this paper is the first version, CP150637.1. The raw ONT and Illumina sequence reads have been deposited in NCBI Sequence Read Archive (SRA) under BioProject accession no. PRJNA1092626 as run accessions SRR29085936 and SRR29085937, respectively.
